# Small Oscillatory Accelerations, Independent of Matrix Deformations, Increase Osteoblast Activity and Enhance Bone Morphology

**DOI:** 10.1371/journal.pone.0000653

**Published:** 2007-07-25

**Authors:** Russell Garman, Clinton Rubin, Stefan Judex

**Affiliations:** Department of Biomedical Engineering, State University of New York at Stony Brook, Stony Brook, New York, United States of America; Massachusetts Institute of Technology, United States of America

## Abstract

A range of tissues have the capacity to adapt to mechanical challenges, an attribute presumed to be regulated through deformation of the cell and/or surrounding matrix. In contrast, it is shown here that extremely small oscillatory accelerations, applied as unconstrained motion and inducing negligible deformation, serve as an anabolic stimulus to osteoblasts *in vivo*. Habitual background loading was removed from the tibiae of 18 female adult mice by hindlimb-unloading. For 20 min/d, 5 d/wk, the left tibia of each mouse was subjected to oscillatory 0.6 g accelerations at 45 Hz while the right tibia served as control. Sham-loaded (n = 9) and normal age-matched control (n = 18) mice provided additional comparisons. Oscillatory accelerations, applied in the absence of weight bearing, resulted in 70% greater bone formation rates in the trabeculae of the metaphysis, but similar levels of bone resorption, when compared to contralateral controls. Quantity and quality of trabecular bone also improved as a result of the acceleration stimulus, as evidenced by a significantly greater bone volume fraction (17%) and connectivity density (33%), and significantly smaller trabecular spacing (−6%) and structural model index (−11%). These *in vivo* data indicate that mechanosensory elements of resident bone cell populations can perceive and respond to acceleratory signals, and point to an efficient means of introducing intense physical signals into a biologic system without putting the matrix at risk of overloading. In retrospect, acceleration, as opposed to direct mechanical distortion, represents a more generic and safe, and perhaps more fundamental means of transducing physical challenges to the cells and tissues of an organism.

## Introduction

Most, if not all, eukaryotic cells are sensitive to mechanical signals, and it has generally been assumed that the magnitude of the cellular response will correspond to the magnitude of the deformation. This is particularly true in bone tissue where the mineralized matrix seemingly protects the resident cell population from high levels of deformation, and thus higher loads are considered necessary to transduce load information to osteoblasts and osteocytes. The mechano-responsiveness of bone was recognized as early as the 16^th^ century [1], and since, it has been presumed that a threshold of 0.1% strain would have to be exceeded to become anabolic [2], while strains below this level of deformation were considered insufficient to retain tissue morphology and thus would be permissive to catabolism [3], [4].

Contrasting with this *more is better* principle, recent work suggests that matrix strains two orders of magnitude below this threshold can be anabolic to bone [5], [6]. The anabolic potential of these vibratory mechanical signals that generate matrix deformations of less than 0.001% strain depended on the frequency at which they were applied, with the greatest response arising within the range of 20–100 Hz [7], [8]. The means by which such low-level mechanical signals can be anabolic to a tissue such as bone is not clear. If cortical matrix deformations of less than 0.001% strain, measured at the periosteum, were transduced directly to the resident osteoblast or osteocyte population, the deformation of the cell itself would be less than one Angstrom. Given that such deformations may be too small to be recognized by cells [9], [10], byproducts of matrix deformation, such as fluid flow induced shear stresses, streaming potentials, fluid drag on pericellular processes, or enhanced nutrient transport, may contribute to a cell's responsiveness to mechanical signals [11], [12]. Yet even these alternative pathways are dependent on matrix deformation and therefore will be very small in magnitude during low-level mechanical stimulation.

In contrast to a matrix deformation dependent pathway for mechanotransduction, the frequency sensitivity of the adaptive system points towards a more fundamental, perhaps unrecognized, pathway by which physical signals interact with the tissues and cells. Indeed, a mechanism that would allow a cell to sense mechanical signals directly without reliance on matrix strain would obviate the need for compensatory tissue-level amplification mechanisms [9], reduce complexity in the system, and may provide cells with mechanical information without the potential for damaging the surrounding tissue. Our hypothesis is that the physical acceleration of a cell may present such a signal which can transmit physical challenges to a receptive cell population in an efficient and safe manner [13].

In the study reported here, bone's habitual loading environment was removed, and very small-amplitude oscillatory accelerations were applied *in vivo*, inducing motion but no direct deformation. It was hypothesized that the resident cell population would be able to sense and respond to these acceleratory signals as anabolic and that the product of the cell response would culminate in a morphology with enhanced tissue quantity and architecture.

## Materials and Methods

### Experimental design

All procedures were reviewed and approved by the Institutional Animal Care and Use Committee (IACUC). Adult (19 wk) female BALB/cByJ mice were used for all experiments. To suppress the potential confounding interference of the experimental physical stimulus with that induced by normal functional load bearing, the first group of 18 mice were tail-suspended for 21 d such that their hindlimbs failed to make contact with the ground [14]. For 20 min each day, each experimental mouse was removed from the hindlimb unloading apparatus and anesthetized with a gaseous 2% isoflurane/oxygen mix. During transport and induction of anesthesia, the hindlimbs of the mouse were prevented from making contact with the ground. The left tibia was attached to an acceleration device [13] that applied sinusoidal oscillatory accelerations at a frequency of 45 Hz and peak accelerations of 0.6 g in the longitudinal direction of the bone (ACC). The right tibia was not subject to any physical intervention and served as an intra-animal contralateral control (CTR). A second group of mice (n = 9) was included to test whether the attachment of the tibia to the acceleration device by itself may induce cellular and morphological changes in the unloaded tibia. These sham-loaded disuse mice were subjected to identical experimental conditions as the first group with the only difference that the left tibia was attached to a vibration device for 20 min/d that did not oscillate (SHAM). As in the first group, the right tibia served as internal contralateral control (SHAM-CTR). Neither hindlimb suspension nor oscillatory motions were applied to 18 mice which were allowed normal cage activity and served as age-matched controls (AGE-CTR).

In vivo micro-computed tomography (VivaCT, Scanco Medical, SUI) was used to quantify bone morphology and micro-architecture in left and right tibiae of all experimental-, sham-, and age-matched control mice. Baseline scans were performed one day prior to commencing the respective protocol (day −1) and on the day of sacrifice (day 21). To aid in the determination of indices of bone formation, calcein injections were administered to all mice on days 9 and 19 (15 mg/kg, i.p.). To minimize the technical complications inherent in determining bone formation and bone resorption activity on sections of the same bone, these analyses were performed on different subsets of mice. Nine tibiae of the experimental mice and eight tibiae of the age-matched control mice were prepared to allow for the analysis of bone formation (undecalcified sections for bone histomorphometry), while the remaining tibiae were used to quantify bone resorption (decalcified sections for TRAP staining).

### Application of high-frequency accelerations

An acceleration device comprising a transducer and function generator delivered high-frequency sinusoidal accelerations to the tibia along its longitudinal axis. Similar to previous experiments using whole body vibration in mice [15], the frequency was set at 45 Hz, producing peak accelerations of 0.6 g and peak-to-peak plate displacements of approximately 76 microns. Just prior to the acceleration input, the mouse was anesthetized and placed in a supine position. Coupling of the transducer to the tibia, and thus transmittance of the acceleratory motion, was achieved by securing the distal portion of the tibia to the plastic extension of the actuator with two pieces of soft rubber. We have previously shown that this manner of attachment provides excellent transmittance of the physical signal and that the resultant peak strain on the periosteal surface of the proximal tibia falls below 0.0003% strain [13], more than three orders of magnitude below the peak strains measured in bone during strenuous activity [16].

### Micro-computed tomography

Trabecular and cortical bone morphology of the tibia was assessed from the *in vivo* microCT scans using an isometric voxel size of 11.5 µm for trabecular bone and 21 µm for cortical bone. Metaphyseal trabecular bone of the proximal tibia was quantified in a region located between 300 µm and 600 µm distal from the growth plate. For cortical bone, a mid-diaphyseal region spanning 300 µm centered on the midsection of the tibia. Noise in the reconstructed images was minimized using a 3D Gaussian filter for which “sigma” and “support” were set at 0.5 and 1, respectively. Bone was segregated via thresholding routines as described previously [17]. For trabecular bone, bone volume fraction (BV/TV), connectivity density (Conn.D), the structural model index (SMI), trabecular number (Tb.N), trabecular thickness (Tb.Th), and trabecular separation (Tb.Sp) were determined. Assessment of cortical bone morphology comprised cortical area (Ct.Ar), as well as endocortical envelope (En.Ev) and periosteal envelope (Ps.Ev) areas.

### Histomorphometry

Indices of bone formation were assessed in metaphyseal trabecular and diaphyseal endocortical bone (no labeling was evident on the periosteal mid-diaphysis). Following euthanasia, the left and right tibia were fixated in 10% formalin and dehydrated in isopropyl alcohol. The samples were infiltrated with a series of three solutions comprising methyl methacrylate (85%), n-butyl phthalate (15%), and benzoyl peroxide (0 g/100 ml, 1 g/100 ml, 2 g/100 ml), and then embedded in polymerized methyl methacrylate. Coronal 7 µm sections were cut from the metaphysis with a rotary microtome (Model 2165, Leica, Wetzlar, Germany) while transverse 40 µm sections were cut from the mid-diaphysis with a diamond wire saw (Model 3241, Well Diamond Wire Saws, Inc., Norcross, GA). Calcein labels were traced using standard software (Osteomeasure, OsteoMetrics Inc., Atlanta, GA) that computed the amount of single labeled surface (sLS/BS), double-labeled surface (dLS/BS), mineralizing surface (MS/BS), mineral apposition rate (MAR), and bone formation rate (BFR/BS) [18].

### TRAP staining

Resorption activity was assessed in trabecular bone of the metaphysis by staining for tartrate resistant acid phosphatase (TRAP). Following euthanasia, each tibia was fixed in 10% formalin, decalcified in 2.5% formic acid, dehydrated in increasing concentrations of ethyl alcohol (70%, 95%, 100%), and then embedded in glycol methacrylate (JB-4 Embedding Kit, Polysciences, Inc.). Coronal 7 µm sections of the proximal tibia were cut on a rotary microtome (Model 2165, Leica, Wetzlar, Germany) and stained for TRAP activity, as described previously [6]. To enhance the contrast between the TRAP stain and the bone surface, sections were counterstained with methyl green. The ratio of osteoclast surface to bone surface (Oc.S/BS) was computed (Osteomeasure).

### Statistics

Differences in histologic and morphometric indices between the left and right tibiae within the same group of mice were determined with non-parametric Wilcoxon tests. Wilcoxon tests were also used to compare microCT based bone morphology between baseline (day −1) and completion (day 21). Differences between AGE-CTR, CTR, and ACC tibiae or SHAM, SHAM-CTR, and CTR tibiae were evaluated by Kruskal-Wallis tests with Dunn post-hoc tests. SigmaStat for Windows 3.10 (Systat Software Inc., Richmond, California) was used for all statistical comparisons. Statistical significance was set at 5%. All data were presented as mean±standard deviation, unless specified otherwise.

## Results

### Baseline morphology

At baseline, there were no differences in trabecular or cortical bone morphology between tibiae pertaining to the different groups used in this study (**Table 1**). There were also no internal baseline differences in metaphyseal or diaphyseal bone morphology between left and right hindlimbs of age matched control mice and, therefore, the average of the two limbs for any given index was used for further analysis.

**Table 1:** Baseline Metaphyseal and Diaphyseal Bone Morphology of the Tibia for the Five Different Groups.

Tibia	BV/TV [1]	Conn.D [1/mm^3^]	Tb.N [1/mm]	Tb.Th [µm]	Tb.Sp [µm]	Ct.Ar [mm^2^]	En.Ev [mm^2^]	Ps.Ev [mm^2^]
CTR	0.19±0.05	103.8±32.8	5.26±0.77	56±3	203±26	0.65±0.04	0.22±0.03	0.91±0.06
ACC	0.20±0.04	117.3±38.1	5.44±0.76	56±2	195±27	0.65±0.04	0.23±0.03	0.93±0.06
SHAM-CTR	0.22±0.04	137.8±64.1	5.63±0.83	57±3	185±24	0.65±0.03	0.23±0.04	0.92±0.07
SHAM	0.23±0.04	138.9±45.6	5.87±0.73	56±3	178±18	0.65±0.04	0.23±0.03	0.94±0.07
AGE-CTR	0.21±0.04	124.7±29.2	5.53±0.57	55±3	190±19	0.64±0.03	0.22±0.03	0.91±0.07

Data are expressed as mean±SD.

METAPHYSIS – BV/TV: bone volume fraction; Conn.D: connectivity density; Tb.N: trabecular number; Tb.Th: trabecular thickness; Tb.Sp: trabecular separation. DIAPHYSIS – Ct.Ar: cortical area; En.Ev: endocortical envelope area; Ps.Ev: periosteal envelope area.

### Acceleration effects on bone formation, resorption, and morphology

**Figure 1:**
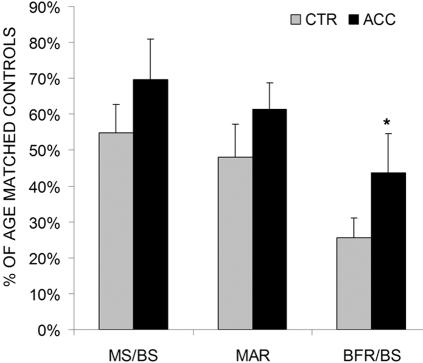
Mean (+SE) mineralizing surfaces (MS/BS), mineral apposition rates (MAR), and bone formation rates (BFR/BS) measured in trabecular bone of the tibial metaphysis of control (CTR) and accelerated tibiae (ACC).

In trabecular bone of the metaphysis, the application of very small amplitude, high frequency accelerations for 20 min/d over a 3 wk period was associated with 70% greater (p<0.05) bone formation rates when compared to contralateral control tibae (**Fig. 1**). Greater BFR/BS were the result of both greater mineralizing surfaces (27%, p = 0.21) and greater mineral apposition rates (28%, p = 0.13). Bone resorption activity was assessed in this region as the ratio of those surfaces that are actively resorbing to the total available trabecular bone surface. The amount of resorptive activity in CTR mice (35.3±7.6%) was greater than in ACC mice (32.5±4.1%) but this difference was statistically not significant.

**Figure 2:**
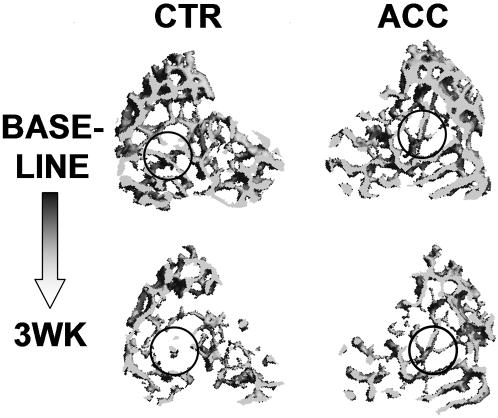
3D reconstructed images of metaphyseal trabecular bone from a tibia that was subjected to short durations of sinusoidal accelerations (ACC, right panel) and its contralateral control (CTR, left panel) at baseline (top row) and at completion of the 3 wk protocol (bottom row). The greater tissue quantity and quality in the accelerated tibia resulted from an enhanced preservation of tissue, as emphasized in the circled regions, during the 3 wk unloading period.

**Figure 3:**
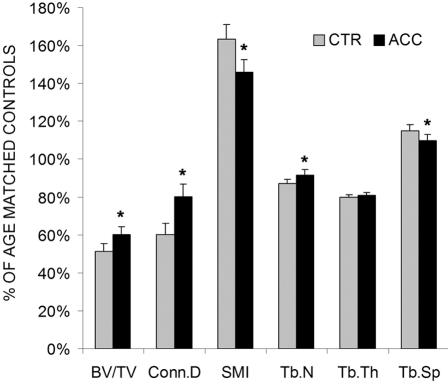
Mean (+SE) bone volume fraction (BV/TV), connectivity density (Conn.D), structural model index (SMI), trabecular number (Tb.N), trabecular thickness (Tb.Th), and trabecular separation (Tb.Sp) of control (CTR) and accelerated tibiae (ACC). Values are expressed as a percentage of their normal weight-bearing age-matched controls. *: p<0.05 between CTR and ACC.

Trabecular bone morphology and micro-architecture were also sensitive to the application of low-amplitude, high-frequency oscillatory accelerations (**Fig. 2**). Metaphyseal bone volume fraction (17%, p = 0.003), connectivity density (33%, p = 0.004), and trabecular number (5%, p = 0.04) were greater in accelerated bones while the structural model index and trabecular separation were 11% (p = 0.01) and 5% (p = 0.04) less (**Fig. 3**). In contrast to the beneficial response measured in trabecular bone, no effects of oscillatory accelerations were seen at 3 wk in the cortical bone of the mid-diaphysis (**Table 2**).

**Table 2:** Histomorphometric and Morphological Indices of Diaphyseal Cortical Bone in Control (CTR), Accelerated (ACC), and Age-Matched Control (AGE-CTR) Tibiae.

	MS/BS [%]	MAR [µm/d]	BFR/BS [µm^3^/µm^2^/yr]	Oc.S/BS [%]	Ct.Ar [mm^2^]	En.Ev [mm^2^]	Ps.Ev [mm^2^]
CTR	6.2±2.6^a^	0.3±0.1	6.4±2.9^a^	12.3±3.7	0.64±0.04	0.23±0.03	0.91±0.06
ACC	6.4±4.7^a^	0.3±0.1	6.8±5.4^a^	13.3±7.2	0.65±0.04	0.24±0.03	0.93±0.06
AGE-CTR	25.7±13.1	0.4±0.2	44.0±30.2	6.7±3.5	0.66±0.04	0.22±0.03	0.93±0.07

Data are expressed as mean±SD.

MS/BS: mineralizing surface; MAR: mineral apposition rate; BFR/BS: bone formation rate; Oc.S/BS: osteoclast surface; Ct.Ar: cortical area; En.Ev: endocortical envelope area; Ps.Ev: periosteal envelope area.

a p<0.05 vs AGE-CTR.

### Comparison to sham controls

Sham control animals were included in the experimental design to confirm that the attachment of the actuator apparatus itself was not responsible for any measurable effect and that the unilateral application of the stimulus did not produce a systemic effect. To address the former, there were no significant differences in any trabecular or cortical morphological parameter between sham-stimulated limbs (SHAM) and their contralateral controls (SHAM-CTR) (**Table 3**). In addressing the latter, all morphological indices were similar between SHAM, SHAM-CTR, and CTR tibiae (**Table 3**).

**Table 3:** Metaphyseal and Diaphyseal Bone Morphology of the Four Different Control Groups at Completion of the Protocol.

Tibia	BV/TV [1]	Conn.D [1/mm^3^]	Tb.N [1/mm]	Tb.Th [µm]	Tb.Sp [µm]	Ct.Ar [mm^2^]	En.Ev [mm^2^]	Ps.Ev [mm^2^]
AGE-CTR	0.20±0.04^a^	97.61±16.1^a^	5.08±0.49^a^	56±3^a^	205±20^a^	0.66±0.04	0.22±0.03	0.93±0.07
CTR	0.10±0.03	58.7±24.5	4.43±0.5	45±4	236±28	0.64±0.04	0.23±0.03	0.91±0.06
SHAM-CTR	0.12±0.02	88.3±28.5	4.65±0.33	44±3	223±16	0.65±0.04	0.23±0.04	0.93±0.07
SHAM	0.12±0.03	84.8±22.8	4.71±0.22	43±3	218±10	0.64±0.04	0.24±0.03	0.93±0.07

Data are expressed as mean±SD.

a p<0.05 for AGE-CTR vs CTR.

METAPHYSIS – BV/TV: bone volume fraction; Conn.D: connectivity density; Tb.N: trabecular number; Tb.Th: trabecular thickness; Tb.Sp: trabecular separation. DIAPHYSIS – Ct.Ar: cortical area; En.Ev: endocortical envelope area; Ps.Ev: periosteal envelope area.

### Comparisons to normal age-matched controls

The primary aim of this study was to determine whether low-level accelerations can be sensed by bone in the absence of habitual functional loading. A secondary aim was to investigate the ability of this stimulus to attenuate unloading induced changes in bone's cellular activity and morphology. To this end, data from CTR and ACC tibiae were compared to normal age-matched controls (AGE-CTR). Compared to trabecular MS/BS (18.2±3.7%), MAR (1.2±0.1 µm/d), and BFR/BS (83.1±17.5 µm/yr) of age-matched controls, the removal of weight bearing (CTR) significantly (p<0.05) reduced indices of bone formation, as indicated by the 45% lower MS/BS, 52% lower MAR, and 74% lower BFR/BS (**Fig. 1**). In ACC tibiae, these differences were attenuated to 30% (MS/BS), 38% (MAR), and 56% (BFR/BS). Osteoclast surface (Oc.S/BS) was significantly greater (p<0.05) in both CTR (68%) and ACC (55%) tibiae than in AGE-CTR bones.

Compared to age matched controls at 3wk, trabecular bone of CTR tibae was characterized by significantly lower BV/TV (49%), Conn.D (40%), Tr.N (13%), Tr.Th (20%) while SMI (63%), and Tb.Sp (15%) were significantly greater. With the application of short bouts of accelerations, these relative differences to normal controls decreased to 40% (BV/TV), 20% (Conn.D), 6% (Tr.N), 19% (Tr.Th), 46% (SMI), and 10% (Tb.Sp) (**Fig. 3**). While bone geometry in the cortical diaphysis was not significantly different between accelerated limbs and their contralateral controls at completion of the protocol, a longitudinal analysis of the *in vivo* microCT scans between baseline and 3wk showed that cortical area in CTR tibiae decreased significantly by 2% (p = 0.009) during the experimental period. In contrast, ACC tibiae did not lose significant amounts of bone. Both groups, however, were significantly different from AGE-CTR which gained 2% (p = 0.003) in cortical area over the 3wk experimental period.

## Discussion

Many biologic systems have the ability to perceive and respond to functional challenges by adapting, but the physical signal which dominates the regulatory input has been difficult to identify. Bone, as an example of an adaptive tissue, can readily adapt to new functional challenges by either increasing bone mass in response to increased demand, or by reducing bone mass in response to disuse. Considering the sensitivity of the skeletal system, however, it is puzzling that such highly orchestrated responses are associated with relatively small deformations. Even during extremely strenuous activities, peak strains in the appendicular skeleton rarely exceed 0.3% strain [19], [20], despite accelerations in excess of 10g [21], [22]. Meanwhile, strain in cartilage can reach 50% [23], while ligaments and tendons typically reach 5–10% deformation [24], [25]. In contrast to a diverse range of strains in the connective tissues, all are subject to similar accelerations and decelerations, pointing towards the possibility of acceleration serving as a more generic physical signal to control the adaptive response. Here, oscillatory accelerations resulted in greater bone formation in trabecular compartments and in enhanced bone morphology. These in vivo data indicate that extracellular matrix strains are not essential for the transduction of mechanical stimuli to tissues, and that cells may have the innate ability to sense acceleration directly.

**Figure 4:**
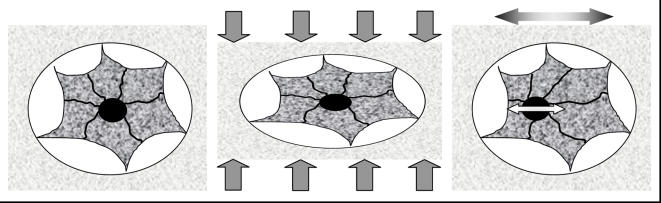
Osteocyte sitting in a lacuna within the matrix (left panel). The nucleus is coupled to the membrane by the cytoskeleton. Upon the application of large loads, the matrix strains and distorts the osteocyte (central panel). These large distortions result in the cytoskeleton pulling on the nucleus, and stimulating transcriptional activity. While this can stimulate a biologic response, it does so at risk of damaging the matrix. Upon the application of sinusoidal accelerations, the bone matrix moves forward and back (or up and down). The cell within the lacunae will oscillate out of phase with the matrix and the nucleus will oscillate out of phase with the cell body, causing the cytoskeleton to pull on the nucleus in the absence of matrix distortion (right panel). In this scenario, accelerations can alter biologic activity in the absence of direct loading, with the potential to distort the cell much greater than with direct loading of the calcified matrix.

The mechanisms by which physical signals are sensed by a cell have routinely focused on specific components of the mechanical information resulting from load, such as stretch or shear of the cell membrane. The *in vivo* sensitivity of bone cells to even extremely small accelerations reported here suggests an alternative and efficient pathway for the transduction of physical signals to the transcriptional machinery of the nucleus (**Fig. 4**). Teleologically, accelerations represent a fundamentally efficient means of delivering regulatory physical information to the cell, and can be readily achieved even in the absence of matrix strains and large cellular deformations. Because the stiffness of the extracellular matrix across different tissues such as skin, ligament, or bone can differ by orders of magnitude, the ubiquitous nature of accelerations throughout the body could also provide a unifying principle by which adaptive cell systems are subject to, and respond to a given mechanical input (e.g., the transmission of a given deceleration on heel strike during locomotion would represent a generic signal to all tissues in the weightbearing musculoskeletal system). Of course, the well documented response of bone cells and tissue to large deformations in the virtual absence of significant accelerations (5–8 orders of magnitude lower than those employed here) [26], [27] emphasizes that there are a number of pathways by which cells can process physical input.

Recent evidence has demonstrated the sensitivity of the musculo-skeletal system to extremely low-level whole body vibrations [28], [29], with the anabolic response assumed to be a product of the forces on, and resultant deformations to, the matrix. Here, a similar frequency and acceleration magnitude also served as a stimulatory signal, achieved in the absence of weightbearing, and thus the absence of directly applied matrix deformation. Even though different experiment designs preclude direct comparisons, it is unlikely that the matrix deformations during whole body vibrations are critical for transducing the physical signal to the regulatory machinery of the cell. It is important to note that, despite the unconstrained nature of the motion applied here, high-frequency accelerations did not entirely eliminate strains in the matrix because the acceleration of a mass will produce a local force. The matrix deformations induced by these forces are extremely small, however, and on the same order as those produced by postural stability [13], [30]. These data suggest that bone can directly benefit from oscillatory motions and indicates, albeit indirectly, that mechanical strain is not necessarily the driving force controlling the adaptive responses in bone. Consistent with such a hypothesis, recent data indicate that, in vitro, bone cells can detect physical stimuli directly in the absence of significant substrate deformations [31].

If acceleration of the nucleus contributes to the recognition of functional load-bearing, it may be surprising that cortical and trabecular bone did not respond similarly. However, this regional specificity has been observed in other studies [32], where the predominant cellular and morphological effects were observed in trabecular bone, suggesting that additional factors play a role in regulating cellular activity. Such factors may be related to the different metabolic rates or surface to volume ratios between trabecular and cortical bone.

This mouse model benefits from the uni-lateral application of the acceleration stimulus to a single limb, allowing the use of an internal contra-lateral control limb and thereby reducing the potential of confounding factors and elevating statistical power. Comparisons between the experimental and sham-control mice confirmed that neither the attachment of the tibia to the device, nor the presence of electromagnetic fields from the transducer were confounding variables, and supports the conclusion that altered regulation of cellular activity and bone morphology were due to the accelerations itself. The use of a genetic mouse strain that is exceptionally sensitive to unloading, while its sensitivity to low level, high frequency loading is only average [15], [17], may have underestimated the efficacy of the stimulus in attenuating bone loss during disuse and a different response may be observed in those mouse strains or animal species that are preferentially sensitive to increased, rather than decreased functional challenges.

High frequency accelerations, applied in the absence of habitual background loading were readily sensed by bone cells and resulted in trabecular bone formation and morphology distinct from disuse alone. Similar to other physical signals that do not necessarily require matrix deformation to be influential in the adaptive process, such as pulsed electromagnetic fields or ultrasound [33], a response was achieved despite the short daily duration of the stimulus and the extremely low amplitude. Thus, accelerations may be a fundamental, efficient, and generic means of inputting functional challenges into an adaptive system – without exposing the matrix to mechanical challenges. The efficacy of the signal to prevent and treat low bone mass in patients confined to bedrest, suffering from spinal cord injuries or osteoporosis, or inflicted with neuromuscular diseases will require further investigation.
